# First-in-human use of ^11^C-CPPC with positron emission tomography for imaging the macrophage colony-stimulating factor 1 receptor

**DOI:** 10.1186/s13550-022-00929-4

**Published:** 2022-09-30

**Authors:** Jennifer M. Coughlin, Yong Du, Wojciech G. Lesniak, Courtney K. Harrington, Mary Katherine Brosnan, Riley O’Toole, Adeline Zandi, Shannon Eileen Sweeney, Rehab Abdallah, Yunkou Wu, Daniel P. Holt, Andrew W. Hall, Robert F. Dannals, Lilja Solnes, Andrew G. Horti, Martin G. Pomper

**Affiliations:** 1 Department of Psychiatry and Behavioral Sciences, Johns Hopkins Medical Institutions, Baltimore, MD USA; 2 Russell H. Morgan Department of Radiology and Radiological Science, Johns Hopkins Medical Institutions, Baltimore, MD USA

**Keywords:** Human PET neuroimaging, Microglia, CSF1R, Neuroinflammation, PET

## Abstract

**Purpose:**

Study of the contribution of microglia to onset and course of several neuropsychiatric conditions is challenged by the fact that these resident immune cells often take on different phenotypes and functions outside the living brain. Imaging microglia with radiotracers developed for use with positron emission tomography (PET) allows researchers to study these cells in their native tissue microenvironment. However, many relevant microglial imaging targets such as the 18 kDa translocator protein are also expressed on non-microglial cells, which can complicate the interpretation of PET findings. ^11^C-CPPC was developed to image the macrophage colony-stimulating factor 1 receptor, a target that is expressed largely by microglia relative to other cell types in the brain. Our prior work with ^11^C-CPPC demonstrated its high, specific uptake in brains of rodents and nonhuman primates with neuroinflammation, which supports the current first-in-human evaluation of its pharmacokinetic behavior in the brains of healthy individuals.

**Methods:**

Eight healthy nonsmoker adults completed a 90-min dynamic PET scan that began with bolus injection of ^11^C-CPPC. Arterial blood sampling was collected in order to generate a metabolite-corrected arterial input function. Tissue time-activity curves (TACs) were generated using regions of interest identified from co-registered magnetic resonance imaging data. One- and two-tissue compartmental models (1TCM and 2TCM) as well as Logan graphical analysis were compared.

**Results:**

Cortical and subcortical tissue TACs peaked by 37.5 min post-injection of ^11^C-CPPC and then declined. The 1TCM was preferred. Total distribution volume (*V*_T_) values computed from 1TCM aligned well with those from Logan graphical analysis (t* = 30), with *V*_T_ values relatively high in thalamus, striatum, and most cortical regions, and with relatively lower *V*_T_ in hippocampus, total white matter, and cerebellar cortex.

**Conclusion:**

Our results extend support for the use of ^11^C-CPPC with PET to study microglia in the human brain.

**Supplementary Information:**

The online version contains supplementary material available at 10.1186/s13550-022-00929-4.

## Introduction

Imaging of microglia and their contribution to brain injury and repair across neuropsychiatric conditions has been widely pursued [1, 2]. Radiotracers developed for use with positron emission tomography (PET) can allow researchers to study microglia in their native tissue microenvironment [3–5], which has advantages since these resident immune cells assume different morphologies ex vivo [6]. However, relevant microglial targets such as 18 kDa translocator protein may be expressed on other cell types in human brain [4, 5, 7]. We developed ^11^C-CPPC to image the macrophage colony-stimulating factor 1 receptor (CSF1R), which is expressed almost exclusively by microglia in the brain [8].

CSF1R is a type III tyrosine kinase receptor that is activated by binding of its endogenous ligands, colony-stimulating factor 1 or interleukin 34 [9]. There is no clear human brain region devoid of the receptor, and CSF1R RNA expression has been reported in many brain regions including thalamus, cerebral cortex, cerebellum, white matter, and hippocampus [10]. CSF1R signaling plays a role in the survival, homeostatic functions, and proliferation of microglia, and its expression may be higher in the brains of patients with certain neurologic conditions such as Alzheimer’s disease or amyotrophic lateral sclerosis [11]. Existing and emerging inhibitors of CSF1R may prevent neurodegeneration in models of Alzheimer’s disease [12] and traumatic brain injury [13, 14], which supports further research into the therapeutic potential of CSF1R antagonism in select neurologic conditions.

^11^C-CPPC [8] is an isotopolog of the selective CSF1R inhibitor, 5-cyano-N-(4-(4-methylpiperazin-1-yl)-2-(piperidin-1-yl)phenyl)furan-2-carboxamide [15]. Unlabeled CPPC has beneficial properties to promote blood–brain barrier permeability such as ideal lipophilicity [calculated partition coefficient (clogD7.4) of 1.6] and molecular mass of 393 Da [8]. We recently demonstrated high, specific uptake of ^11^C-CPPC in murine and nonhuman primate models of neuroinflammation [8] and now present its first-in-human use with PET. This study assessed the pharmacokinetic behavior of ^11^C-CPPC in the brains of eight healthy individuals. Estimates of regional kinetic parameters and total distribution volume (*V*_T_) values were generated using pharmacokinetic modeling methods with a metabolite-corrected arterial input function, and the different model fits were compared.

## Materials and methods

### Human subjects

The Johns Hopkins Institutional Review Board approved this study, and all research participants provided written, informed consent. Adult (≥ 18 years of age) individuals completed a screening clinical research interview and laboratory testing (blood counts, metabolic panel, coagulation studies, pregnancy testing when applicable) with electrocardiogram and urine toxicology. Eligible participants were assessed as being in stable health with no clinical abnormality on the screening assessment and structural magnetic resonance imaging (MRI). A Johns Hopkins faculty neuroradiologist reviewed each structural MRI. Exclusion criteria included use of cannabis, nicotine, or other recreational substances in the past six months (assessed by self-report and urine toxicology), prescribed or over-the-counter anti-inflammatory medication within two weeks prior to the PET, acute onset or exacerbation of illness/infection in the past month, contraindication to MRI, or contraindication to PET imaging with an arterial line.

### Human brain imaging

#### *Synthesis of *^*11*^*C-CPPC*

^11^C-CPPC was synthesized as described by Mathews et al. [16] at the Johns Hopkins PET Radiotracer Center. The synthesis and all major quality measures (including radiochemical purity > 95%) were in compliance with standard good manufacturing practice for PET radiotracers. Molar activity of ^11^C-CPPC at the time of injection was 316.8 ± 138.8 GBq/µmol. The mean administered mass and radioactivity of ^11^C-CPPC were 1.2 ± 1.1 μg (range 0.55–3.72 μg) and 651.4 ± 62.8 MBq (range 542.8–736.7 MBq), respectively.

#### MRI acquisition and regions of interest

A T1-weighted Magnetization-Prepared Rapid Gradient-Echo (MP-RAGE) sequence with 0.75 × 0.75 × 0.8 mm voxel size was acquired from each participant using a 3 Tesla MRI system (Siemens MAGNETOM Prisma, Malvern, PA, USA or Philips Achieva, Best, Netherlands). MRI data were segmented using the FreeSurfer image analysis suite (http://surfer.nmr.mgh.harvard.edu/). Ten regions of interest (ROIs) were selected: total white matter, thalamus, striatum, hippocampus, as well as cerebellar, temporal, occipital, cingulate, frontal, and parietal cortices (Additional file 1: Fig. S1).

#### PET acquisition and reconstruction

Before the PET, each participant underwent fitting of a thermoplastic facemask, as well as insertion of a radial arterial catheter and intravenous catheter. The thermoplastic facemask was used for head fixation during the PET to minimize head motion. An attenuation map was estimated from a 6 min transmission scan that was performed with a ^137^Cs point source prior to the emission scan. Each emission scan started at the time of bolus intravenous injection of ^11^C-CPPC, with continuous list mode data collection for 90 min on a High Resolution Research Tomograph (Siemens Healthcare, Knoxville, TN) [17]. Imaging data were reconstructed using the iterative ordinary-Poisson ordered-subset expectation–maximization algorithm (6 iteration and 16 subsets, 2 mm post-smoothing) and were corrected for decay, attenuation, random activity, and scatter [18]. The reconstructed data were binned into 30 frames (four 15 s, four 30 s, three 1 min, two 2 min, five 4 min, and twelve 5 min). The reconstructed image volume spanned 31 cm × 31 cm transaxially and 25 cm axially, with image matrix of 256 × 256 × 207 voxels (voxel size of 1.22 × 1.22 × 1.22 mm).

#### Plasma acquisition

Arterial whole blood samples were collected manually over the course of the emission scan. First, 7 ml of blood were drawn for the protein binding sample and 4 ml of blood were drawn for the pre-injection/background high-performance liquid chromatography (HPLC) measurement. Then, 1 ml blood samples were collected from (a) time of injection to 1.5 min post-injection (p.i.) at a rate as fast as possible (a total of 16–18 samples); (b) 1.5–3 min p.i every 30 s; (c) 3–5 min p.i every 2 min; (d) 5–30 min p.i. every 5 min; and (e) from 30 to 90 min p.i. every 15 min. An additional volume of 3–6 mL was drawn at the 5, 10, 20, 30, 60, and 90 min time points for HPLC measurements. Plasma was immediately isolated from each blood sample using centrifugation before plasma radioactivity was counted in a cross-calibrated gamma well-counter (PerkinElmer 2480 WIZARD2 Automatic Gamma Counter, Shelton, CT). The previously described modified column-switching HPLC method was used to measure the fraction of parent ^11^C-CPPC in plasma at 5, 10, 20, 30, 40, 60, and 90 min p.i. [8]. Metabolite-corrected time-activity curves (TACs) were generated by applying the parent ^11^C-CPPC fraction to the total plasma TACs using linear interpolation (v3.7, PMOD Technologies Ltd., Zurich, Switzerland). Ultrafiltration (Centrifree Ultrafiltration Device, MilliporeSigma, Burlington, MA) was used to measure plasma free fraction (*f*_P_) of ^11^C-CPPC.

#### PET data processing

PET data preprocessing steps as well as the kinetic analyses were conducted using PMOD (v3.7, PMOD Technologies Ltd., Zurich, Switzerland). Post-reconstruction inter-frame motion correction was applied as needed by frame-by-frame matching of dynamic data to a static reference frame generated from the average of the frames corresponding to 30–60 min p.i. PET data were rigidly transformed into MR space by co-registering the mean 30–60-min PET image and each of the 30 motion-corrected PET frames to the T1-weighted MRI image.

#### Derivation of PET rate constants and distribution volumes

Total distribution volume, *V*_T_ [19], for each ROI was derived using the metabolite-corrected arterial input function and compartmental modeling (one-tissue compartment model with 3 parameters, 1TCM; two-tissue compartment model with 4 parameters, 2TCM) or Logan graphical analysis [20]. The delay between the input function and tissue signal was estimated using a fit for the whole brain and then fixed across the ROI fits. Across participants, the mean estimated delay was < 2 s. Cerebral blood volume was set to 5% of brain volume in compartmental models, with *C*_model_(*t*) = (1 − vB)*C_T_(*t*) + vB*C_P_(*t*), wherein *C*_model_ is the modeled curve, vB is blood volume fixed at 0.05, *C*_T_ is the measured tissue activity, and *C*_P_ is measured plasma activity. Nonlinear least squares regression was used to calculate the parameters of the compartmental models and the % standard error (%SE) of each estimated parameter. In Logan graphical analysis, various selections of equilibration time, t*, were evaluated across the ROIs using the 10% max error criterion, with ultimate selection of t* = 30 min.

### Statistics

The compartmental model fits that were applied to the regional TACs were first assessed visually before the relative goodness of fit was assessed using the *F* test [21]. Regional *V*_T_ estimates from variable scan durations were evaluated against those *V*_T_ values estimated from the full 90-min acquisition through data shortening in 5 min intervals down to a 50-min continuous scan duration p.i. For each duration, denoted *X*, relative bias values were expressed as |*V*_TX_ − *V*_T 90 min_|/*V*_T 90 min_. Data are presented as mean ± standard deviation unless otherwise noted.

## Results

### Human subjects

Eight healthy nonsmokers (four males, four females, age range 24–65 years, median 42 years, interquartile range 22 years) underwent PET neuroimaging with ^11^C-CPPC. Participants were of Caucasian (*N* = 4), African-American (*N* = 2), or Asian (*N* = 2) race and had mean body weight of 81.8 ± 17.2 kg. Based on assessments that included monitoring of vital signs, laboratory testing, and electrocardiograms, there were no adverse or clinically detectable pharmacologic effects from injection of ^11^C-CPPC. Scores on assessments probing signs and symptoms of depression, anxiety, and cognitive impairment were consistent with absence of active neuropsychiatric illness (*N* = 8), with mean Hamilton Depression Rating Scale Score of 0.6 ± 0.9, mean Hamilton Anxiety Rating Scale Score of 0.9 ± 1.1, and mean Mini Mental Status Exam score of 29.8 ± 0.5.

### Plasma analysis

**Fig. 1:**
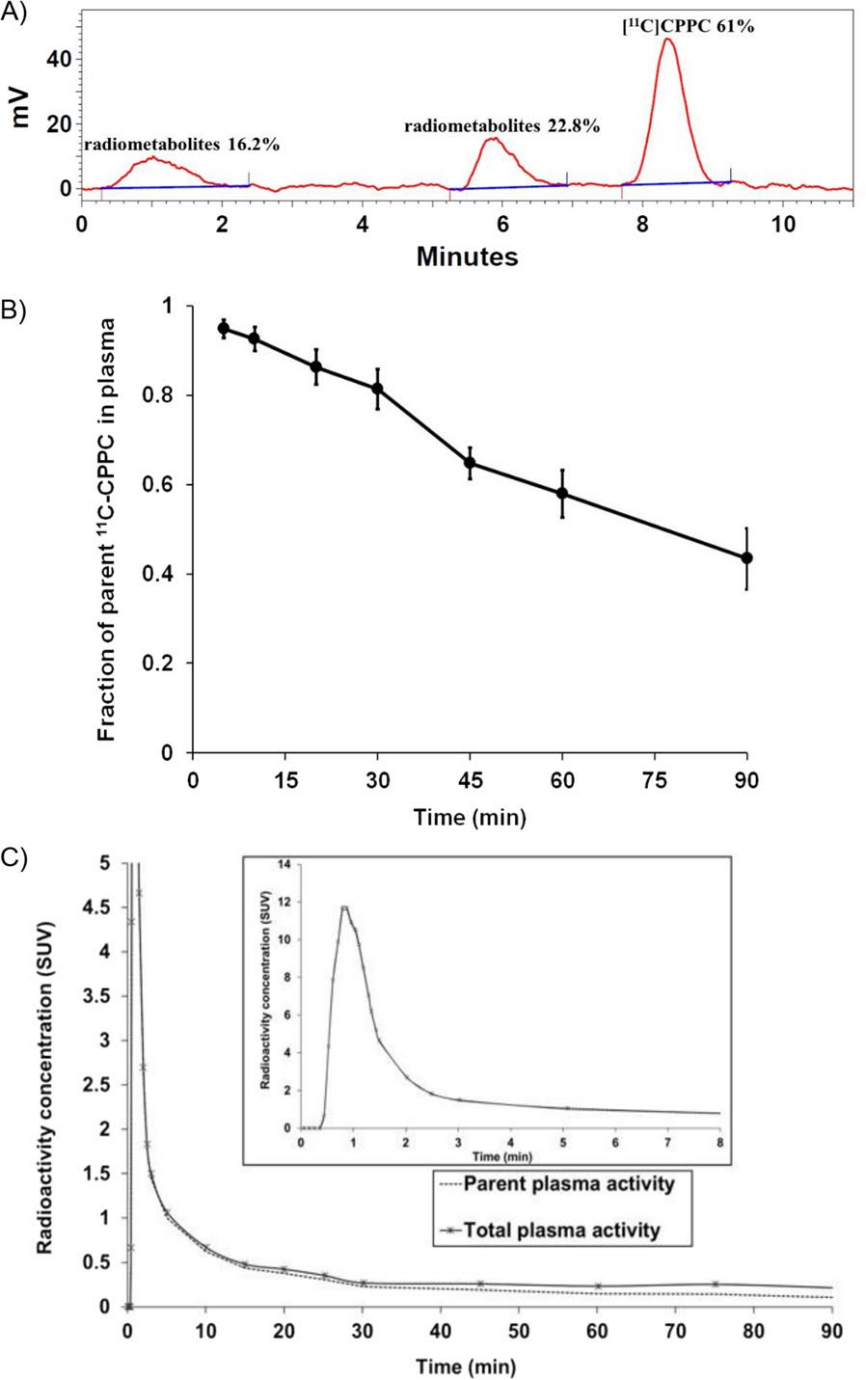
Plasma activity after injection of ^11^C-CPPC. (**A**) HPLC tracing at 60 min post-injection (p.i.) from a representative participant that demonstrates evidence of two radiolabeled metabolites in addition to the peak for parent ^11^C-CPPC. (**B**) Parent fraction (mean with standard deviation) measured at select time points (dots) over 90 min p.i., shown with linear interpolation. (**C**) Radioactivity curves in total plasma (solid line) and the portion of unmetabolized ^11^C-CPPC (dotted line) p.i. Inset shows early time points. Radioactivity is in standardized uptake value, SUV (injected dose per mL plasma normalized to body weight in grams)

HPLC revealed two radiolabeled metabolites that were more polar than the parent, ^11^C-CPPC (Fig. 1A). The parent fraction decreased moderately over the 90 min p.i., with parent ^11^C-CPPC fraction representing 57.9 ± 5.3% and 43.4 ± 6.8% of total plasma activity by 60 min and 90 min, respectively (Fig. 1B). Total plasma activity peaked within 60 s p.i. (Fig. 1C). Measured *f*_P_ was 3.26 ± 0.57%.

### Derivation of kinetic rate constants and *V*_T_

**Fig. 2:**
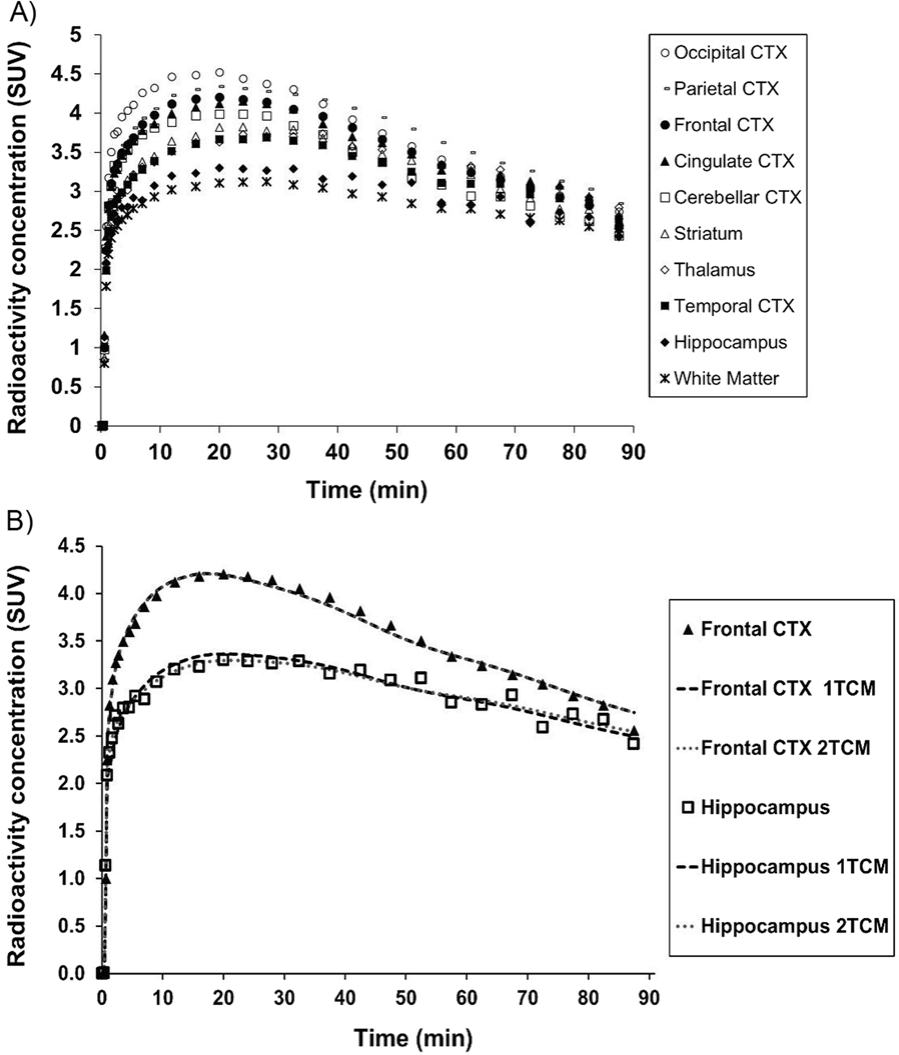
Time-activity curves (TACs) from 90-min ^11^C-CPPC PET data of a representative subject. (**A**) TACs across ten regions of interest (ROIs) are shown as SUV (injected dose per cm^3^ tissue normalized to body weight in grams). (**B**) Visually, the one-tissue compartment model (1TCM) and two-tissue compartment model (2TCM) fit the observed tissue TACs in all ROIs. Observed activity (data in shapes) and model curves (bold dash curve, 1TCM; smaller dash curve, 2TCM) from frontal cortex and striatum are shown. Cortex, CTX

Over the 90-min scan duration, regional radioactivity curves peaked within 37.5 min p.i of ^11^C-CPPC and then declined (Fig. 2A). Highest peak uptake occurred in cortical regions and thalamus, with slightly lower peaks in hippocampus and total white matter.

**Fig. 3:**
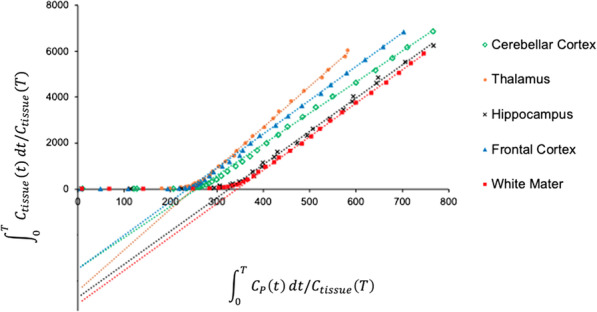
Representative Logan plots from 90-min ^11^C-CPPC PET data across brain regions of interest. Select regional Logan fittings from one healthy participant are shown

**Fig. 4:**
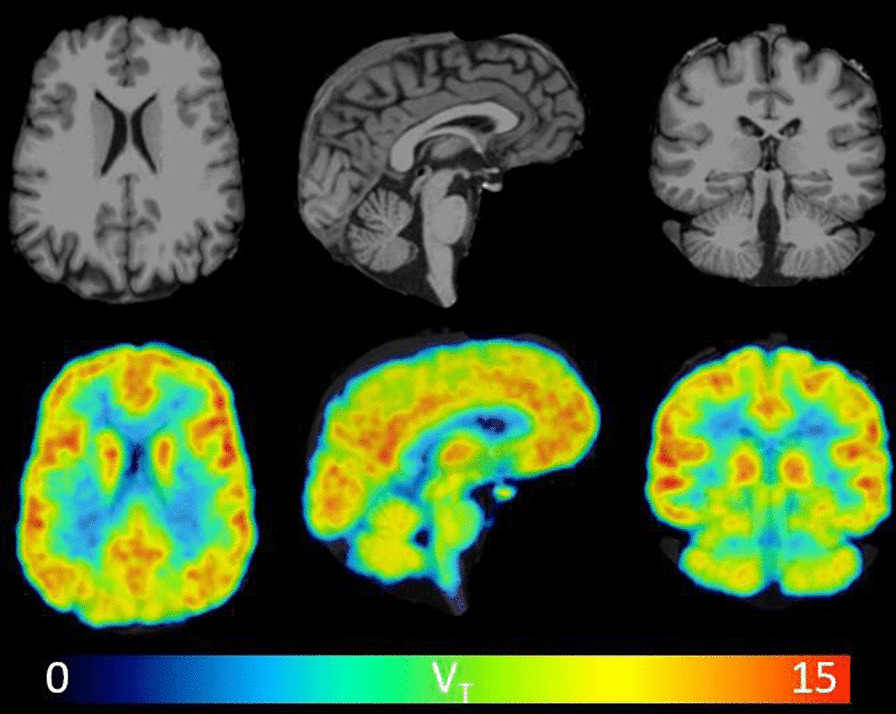
Visual inspection of ^11^C-CPPC total distribution volume (*V*_T_) values across the human brain reveals thalamus as one of the regions of highest binding, and relatively lower binding in cerebellar cortex. Parametric *V*_T_ images and MRI images in three views are shown from a representative healthy participant. *V*_T_ was estimated from 90-min data using Logan graphical analysis with the metabolite-corrected arterial input function. *V*_T_ is in units of mL cm^−3^. The *V*_T_ image was filtered with a 2 mm FWHM 3D Gaussian to reduce noise

**Fig. 5:**
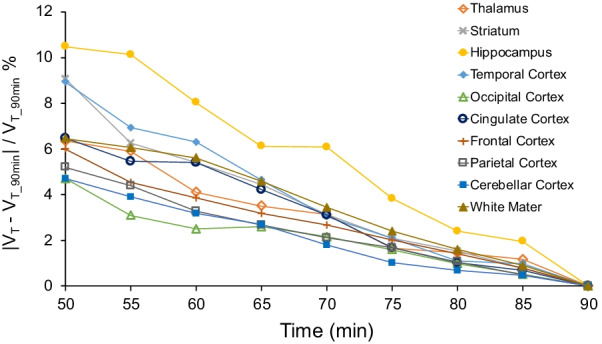
Assessment of the relative stability in ^11^C-CPPC total distribution volume (*V*_T_) values from 90 min of data compared to values produced from truncated scan durations. Ninety-min emission data from eight healthy individuals were shortened in five min intervals down to a minimum 50-min scan duration. Regional *V*_T_ values are in units of mL cm^−3^. The percent absolute difference between the *V*_T_ values from 90 min of data and *V*_T_ values from shortened scan duration is plotted for each of the ten brain regions of interest

Visually, each compartmental model fit the regional TACs well (Fig. 2B). The 1TCM was able to estimate individual parameters (*K*_1_, *k*_2_) and *V*_T_ well (Table 1). The 2TCM estimated *V*_T_ well except for one unstable white matter *V*_T_ estimate from one individual (Table 1). The 2TCM was statistically favored over the 1TCM in 45% of fits by the *F* test. However, the 2TCM did not identify several individual parameters well, as evidenced by *K*_1_ with SE > 10% in 16.3% of fits, and *k*_2_, *k*_3_, and *k*_4_ each with SE > 5% in the majority of fits. After excluding the one unstable white matter *V*_T_ value from 2TCM, regional *V*_T_ from the 1TCM aligned with *V*_T_ estimates from the 2TCM (1TCM *V*_T_ = 1.02 * 2TCM *V*_T_ − 0.47, *R*^2^ = 0.99). The 1TCM was therefore selected as the preferred compartmental model for regional *V*_T_ estimates of ^11^C-CPPC PET data. Since Logan-derived *V*_T_ estimates aligned well with regional *V*_T_ from the 1TCM (Logan *V*_T_ = 0.88 * 1TCM *V*_T_ + 1.07, *R*^2^ = 0.92), Logan analysis was also appropriate for quantitative estimation of *V*_T_. Representative regional Logan plots are shown in Fig. 3. *V*_T_ values (compartmental modeling, Logan) were relatively high in thalamus, striatum and most cortical regions, and lower in hippocampus, white matter, and cerebellar cortex. Parametric images of Logan-derived *V*_T_ are shown in Fig. 4 and Additional file 1: Fig. S2. With the exception of hippocampus, regional Logan-derived *V*_T_ values generated from truncated scan durations down to 65 min were within 5% of the *V*_T_ values obtained using 90-min emission data. In hippocampus, 75 min continuous scans were required to generate *V*_T_ within 5% of the *V*_T_ values obtained using the 90-min data (Fig. 5).

**Table 1:** Kinetic parameters and total distribution volume (*V*_T_) values estimated with the one-tissue compartment model (1TCM), along with *V*_T_ values estimated using two-tissue compartment model (2TCM), and Logan analysis for ^11^C-CPPC PET imaging in humans (*N* = 8)

Region of interest	1TCM			2TCM	Logan
	*K*_1_	*k*_2_	*V*_T_	*V*_T_	*V*_T_
	(mL cm^−3^ min^−1^)	(min^−1^)	(mL cm^−3^)	(mL cm^−3^)	(mL cm^−3^)
Thalamus	0.29 ± 0.04 (0.97)	0.02 ± 0.00 (1.99)	16.6 ± 2.0 (1.34)	16.9 ± 2.1 (1.92)	16.0 ± 2.0 (1.26)
Striatum	0.30 ± 0.04 (0.96)	0.02 ± 0.00 (1.94)	16.2 ± 1.8 (1.30)	16.3 ± 1.9 (3.11)	15.2 ± 1.8 (1.28)
Parietal cortex	0.32 ± 0.03 (0.80)	0.02 ± 0.00 (1.54)	16.1 ± 1.7 (1.02)	16.2 ± 1.7 (1.85)	15.2 ± 1.5 (0.70)
Cingulate cortex	0.31 ± 0.04 (0.92)	0.02 ± 0.00 (1.78)	15.7 ± 2.0 (1.18)	15.8 ± 2.0 (2.68)	14.9 ± 1.9 (1.09)
Temporal cortex	0.26 ± 0.03 (0.92)	0.02 ± 0.00 (1.96)	15.6 ± 1.8 (1.33)	15.7 ± 1.8 (3.04)	14.7 ± 1.6 (1.10)
Frontal cortex	0.32 ± 0.04 (0.85)	0.02 ± 0.00 (1.60)	15.2 ± 1.5 (1.06)	15.3 ± 1.5 (1.31)	14.5 ± 1.5 (0.92)
Occipital cortex	0.31 ± 0.02 (0.90)	0.02 ± 0.00 (1.71)	14.9 ± 1.7 (1.13)	15.0 ± 1.7 (1.92)	14.1 ± 1.5 (0.65)
Hippocampus	0.22 ± 0.02 (1.23)	0.02 ± 0.00 (2.64)	14.1 ± 1.8 (1.80)	14.4 ± 1.8 (2.01)	13.6 ± 1.6 (2.48)
Cerebellar cortex	0.29 ± 0.04 (0.85)	0.02 ± 0.00 (1.58)	13.9 ± 1.4 (1.05)	14.0 ± 1.4 (1.76)	13.2 ± 1.4 (0.61)
Total white matter	0.21 ± 0.02 (1.05)	0.04 ± 0.06 (2.28)	13.6 ± 1.8 (1.55)	14.0 ± 2.0 (1.55)*	13.5 ± 1.8 (1.20)

Presented as Mean ± standard deviation, and mean percent standard error in parentheses

*One individual had poor fit for data from total white matter. The white matter data from this individual were excluded from the reported mean *V*_T_ for this region

## Discussion

Study of the contribution of microglia to onset and course of several neuropsychiatric conditions is challenged by the fact that these brain-resident immune cells often appear and/or react differently in postmortem tissue compared to their native, in vivo microenvironment. Imaging microglia with radiotracers developed for use with PET allows researchers to study these cells in the living human brain, and yet many relevant targets such as the 18 kDa translocator protein are also expressed on non-microglial cells. We developed ^11^C-CPPC to image the CSF1R that is essentially expressed by microglia alone in the brain and present first-in-human evaluation of its pharmacokinetic behavior in the brains of healthy individuals.

The 2TCM estimated *V*_T_ well except for one unstable white matter *V*_T_ estimate from one individual. However, the 2TCM did not identify well several other parameters (*k*2, *k*3, *k*4). The 2TCM was statistically favored over the 1TCM in 45% of fits by the *F* test, and yet regional *V*_T_ from 1TCM aligned with the well-identified *V*_T_ estimates from the 2TCM. Accordingly, the 1TCM was selected as the preferred compartmental model for ^11^C-CPPC PET data. Further, Logan-derived regional *V*_T_ values aligned well with those from the 1TCM, supporting future use of Logan graphical analysis given the relative stability of Logan-derived *V*_T_. Further, the majority of regional Logan-derived *V*_T_ values were well estimated using data from just 65 min acquisition, although the hippocampal region of interest was the exception and required 75 min of continuous data in order to generate *V*_T_ estimates that aligned well with the 90-min scan.

While several other CSF1R-targeting radiotracers have emerged for use with PET, to our knowledge ^11^C-CPPC is the first used in humans. The future use of other CSF1R radioligands may be limited by evidence of poor brain penetrance in mice and nonhuman primates (^11^C-AZ683) [22] or nonspecific binding (^11^C-NCGG401) [23]. Neuroimaging with ^11^C-GW2580 PET has shown promising results in mouse models of neuroinflammation and in nonhuman primates, and yet brain uptake is lower than with ^11^C-CPPC [24]. Future evaluation of ^11^C-GW2580 pharmacokinetics in human brain is anticipated.

CSF1R is expressed by microglia across the brain, and there were no clear regions devoid of ^11^C-CPPC uptake. In alignment with the reported expression pattern of CSF1R across the human brain [10], the highest *V*_T_ values were observed in thalamus, many cortical regions, and striatum, with moderate values in hippocampus and low *V*_T_ in cerebellar cortex. While CSF1R RNA has been reported as high in white matter [10], total white matter *V*_T_ values were low in these first eight healthy individuals. This observation may be due to the defined total white matter ROI in this study since reported RNA expression varies across white matter substructures [10]. The publicly available CSF1R RNA measures in human white matter were also obtained largely from individuals between the ages of 60–94 years, while the median age in the present study was 42 years. Future work should evaluate the CSF1R distribution across white matter regions in postmortem tissue from less elderly individuals and by assessing complementary ^11^C-CPPC *V*_T_ estimates from white matter substructures.

## Conclusion

This study supports further use of ^11^C-CPPC with PET for measuring in vivo the CSF1R in the human brain. ^11^C-CPPC showed high brain uptake across ROIs, and the 1TCM was selected as the preferred compartmental model for estimating regional *V*_T_ from 90-min emission data with a metabolite-corrected arterial input function. Logan-derived regional *V*_T_ estimates aligned well with those from the 1TCM. Given the ability to estimate regional *V*_T_ values well in health, it is also reasonable to pursue future evaluation of ^11^C-CPPC with PET in clinical populations with microgliosis and/or microglial activation driving neurodegeneration.

## Supplementary Information

**Additional file 1: Fig. S1.** Representative views of the ten regions of interest.  **Fig. S2**. ^11^C-CPPC total distribution volume (*V*_T_) values across the human brain (N=8).

## Data Availability

The datasets generated during and/or analyzed during the current study are available from the corresponding author on reasonable request.
