# Supplementary material for: First-in-human use of 11C-CPPC with positron emission tomography for imaging the macrophage colony-stimulating factor 1 receptor
Source: EJNMMI Res. 2022 Sep 30;12:64. doi: 10.1186/s13550-022-00929-4 (PMC9522955; doi:10.1186/s13550-022-00929-4)
Supplement: Supplementary file 1 — Additional file 1: Fig. S1. Representative views of the ten regions of interest. Fig. S2. 11C-CPPC total distribution volume (VT) values across the human brain (N=8). [file 13550_2022_929_MOESM1_ESM.docx]

**Additional file 1**

**Supplemental Figures**

**Fig. S1** Representative views of the ten regions of interest.


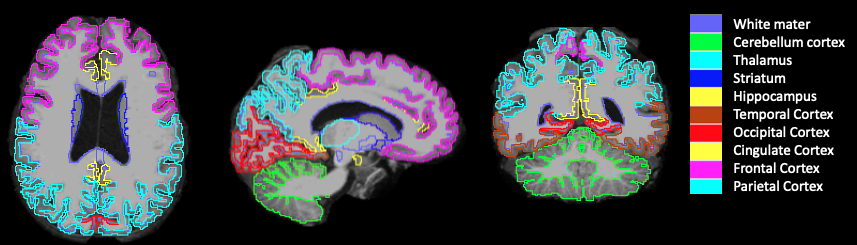


**Fig. S2** ^11^C-CPPC total distribution volume (V_T_) values across the human brain (N=8). Representative skull-stripped MRI (top), ^11^C-CPPC V_T_ map (middle), and fused (lower) data are shown. The individual V_T_ map from each participant was first normalized to the SPM8 standard template and then averaged to obtain the mean ^11^C-CPPC V_T_ map. V_T_ was estimated from 90 min data using Logan graphical analysis with the metabolite-corrected arterial input function. V_T_ is in units of mL cm^-3^.

**
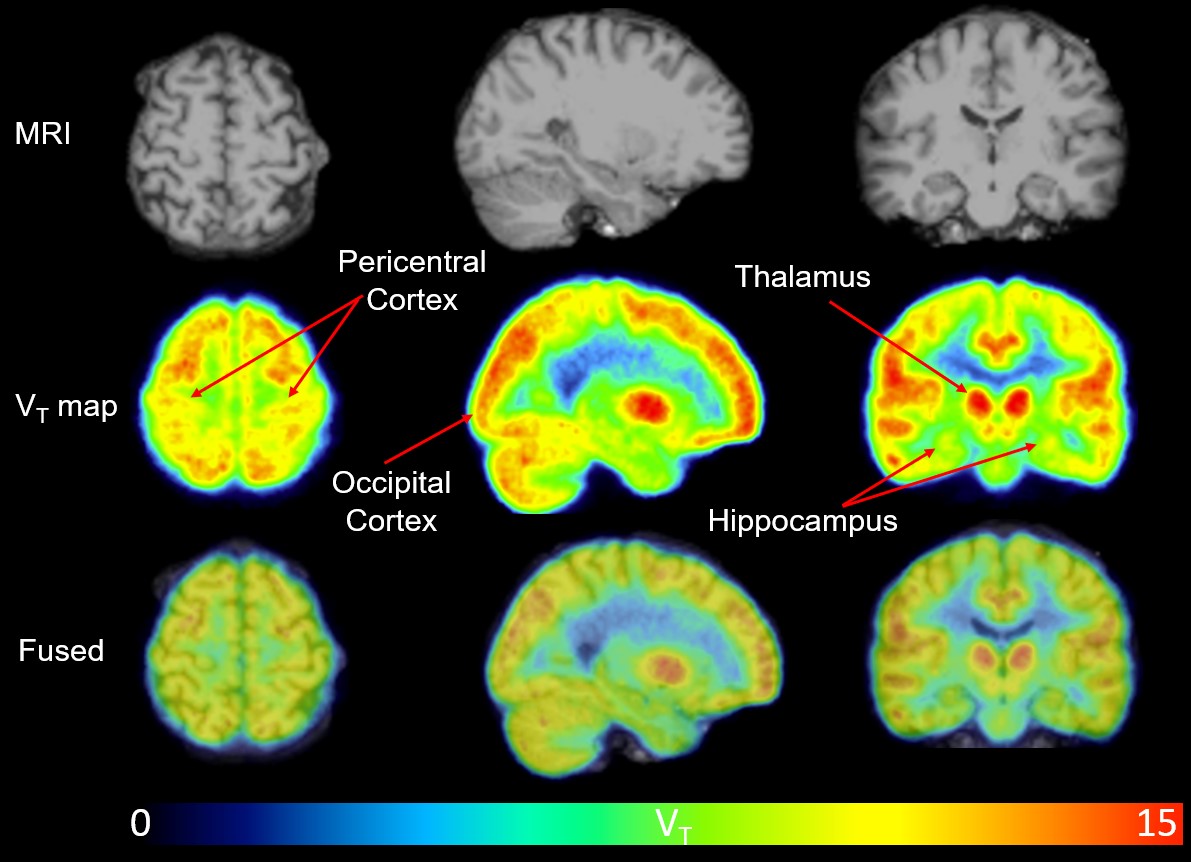
**
